# Effect of Pyrolysis Conditions on Removal of Pb(II) from Aqueous Solution by Biochar Derived from Anaerobically Digested Sewage Sludge Pretreated with nZVI

**DOI:** 10.3390/toxics14030206

**Published:** 2026-02-27

**Authors:** Luiza Usevičiūtė, Vaidotas Danila, Tomas Januševičius, Mantas Pranskevičius

**Affiliations:** Research Institute of Environmental Protection, Vilnius Gediminas Technical University, LT-10223 Vilnius, Lithuania; vaidotas.danila@vilniustech.lt (V.D.); tomas.janusevicius@vilniustech.lt (T.J.); mantas.pranskevicius@vilniustech.lt (M.P.)

**Keywords:** anaerobic digestion, pyrolysis method, thermal hydrolysis, sewage sludge biochar, lead removal, adsorption

## Abstract

This study investigated the ability of anaerobically digested sewage sludge biochar (ADSSBC), pretreated with nanoscale zero-valent iron (nZVI) prior to anaerobic digestion (AD), to remove lead (Pb(II)) ions from aqueous solutions. Batch adsorption experiments were conducted to evaluate the effects of various parameters, including nZVI dosage, O_2_-exclusion method (aluminum foil wrapping or N_2_ purging), pyrolysis temperature (300–800 °C), adsorbent dosage, pH, coexisting ions, contact time, and initial Pb(II) concentration. Experimental data were fitted to adsorption kinetic and isotherm models. The characteristics of nZVI30-ADSSBC-700 before and after Pb(II) adsorption were analyzed using FTIR, SEM–EDS, XPS, and XRD to identify the adsorption mechanisms. The results showed that nZVI addition at 30 mg/g-TS prior to AD significantly enhanced Pb(II) removal efficiency compared with the control. Among the investigated pyrolysis temperatures and O_2_-exclusion methods, the biochar produced at 700 °C using aluminum foil wrapping exhibited the highest Pb(II) removal efficiency (99.4%) at an initial Pb(II) concentration of 200 mg/L. The maximum Langmuir adsorption capacity obtained for this biochar was 139.3 mg/g. The pseudo-second-order kinetic model best described the Pb(II) adsorption kinetics. The investigated models and the results of physicochemical analyses indicated the involvement of both physical and chemical adsorption mechanisms, including surface precipitation, ion exchange, pore filling, and, to some extent, complexation.

## 1. Introduction

The increasing amount of sewage sludge (SS) generated by wastewater treatment plants represents a significant environmental challenge [1]. Sewage sludge poses environmental risks due to the presence of heavy metals (HMs), persistent organic pollutants, and pathogens [2]. Therefore, proper handling and disposal of SS are critical for environmental protection. Common SS management options include anaerobic digestion (AD), thermal treatment, landfilling, and land application as a fertilizer [3]. Anaerobic digestion is widely used because it reduces sludge volume and generates renewable energy by converting organic matter in sludge into methane (CH_4_). Although AD significantly reduces sludge volume, the remaining amount is still significant and requires utilization. Pyrolysis of anaerobically digested sewage sludge (ADSS) offers multiple benefits, including pathogen elimination, sludge volume reduction, and transformation of digestate into a more stable material [4]. The resulting biochar can serve as an adsorptive material capable of removing a wide range of pollutants from wastewater, including HMs. Among HMs, lead (Pb(II)) is particularly hazardous as it poses serious health risks, including anemia and disruption of the digestive system [5]. To date, various methods have been used for the removal of HMs from wastewater, including membrane filtration, chemical precipitation, ion exchange, and adsorption [6]. Adsorption stands out from other methods due to its simplicity of operation and cost-effectiveness, relying on low-cost adsorbents that are now widely used to remove HMs [7]. Plentiful natural materials, such as corn stalk [8], rice straw [9], bamboo [10], and various industrial by-products, including ADSS, can serve as feedstocks for producing such adsorbents due to their high availability and low cost.

Previous studies have shown that sludge-based biochar adsorbents can be effectively used to remove various HMs, including Pb(II), from aqueous solutions [11]. For example, Shao et al. [7] demonstrated that municipal sludge-derived biochar was an efficient adsorbent for the removal of Pb(II), with a maximum adsorption capacity (*q*_max_) of 18.56 mg/g. The key properties of biochar that strongly influence HM removal from water include its specific surface area (SSA), pore structure, abundance of oxygen-containing functional groups, and mineral content. A well-developed porous structure enhances the binding of HMs by increasing the accessibility of sorption sites within the biochar matrix [12]. Oxygen-containing functional groups, such as hydroxyl (–OH) and carboxyl (–COOH) groups, can bind HMs like Pb(II) through electrostatic attraction or complexation mechanisms [13,14]. Sludge-derived biochar is considered an effective material for HM removal due to its high content of mineral phases, such as Fe, Al, and Mn oxides, which provide abundant binding sites and facilitate metal immobilization [15,16,17]. In addition, sludge biochar is rich in phosphates (PO_4_^3−^) and carbonates (CO_3_^2−^), which can react with HMs to form insoluble phosphate and carbonate compounds [7,16,18]. A previous study reported that Pb(II) precipitation with mineral components was the dominant removal mechanism, accounting for 53.5% of total Pb(II) adsorption by AD sludge biochar [15].

The characteristics of sludge-derived biochar can be optimized by selecting appropriate pyrolysis conditions, including temperature and the O_2_-exclusion method. Conventional pyrolysis at elevated temperatures and prolonged residence times (300–1000 °C, >1 h) under limited O_2_ conditions is the most commonly used method to produce biochar from ADSS [2]. Low pyrolysis temperatures result in minimal changes to the surface chemistry of sludge biochar, whereas high-temperature treatment promotes complete carbonization and pore formation due to gas release. Biochar characteristics are also influenced by the gaseous atmosphere during pyrolysis, a factor that has become increasingly important in recent years [19]. A previous study [20] demonstrated that municipal sludge biochar pyrolyzed under CO_2_ at 400 °C and 500 °C exhibited a higher SSA than sludge biochar pyrolyzed under N_2_ at the corresponding temperatures. In contrast, at 600 and 700 °C, higher SSA values were obtained under N_2_ pyrolysis than under CO_2_. Very few studies have performed pyrolysis by wrapping biomass in foil without using a carrier gas [21]. Under these conditions, the foil serves as a physical barrier that restricts O_2_ exposure and protects the material from oxidation during pyrolysis. By adjusting pyrolysis conditions, biochars with distinct physicochemical properties can be produced, which in turn influence their HM adsorption capacity.

To improve the adsorption performance of sludge-based biochars, various modification strategies have been explored, including the loading of biochar with nanoscale zero-valent iron (nZVI) [22]. Numerous studies have incorporated nZVI into sludge-derived biochar to enhance its adsorption capacity for toxic elements and have reported significant improvements in removal performance [23]. For example, Wang et al. [24] compared Cr(VI) removal from an aqueous solution using KOH-activated sludge biochar and the same biochar loaded with nZVI. The results showed that nZVI loading increased Cr(VI) removal rate by 37.78%. In addition, recent studies on nZVI-coated biochars derived from other feedstocks have demonstrated their effectiveness in removing Pb(II). For example, Zhao et al. [9] showed that a calcium alginate-nZVI-rice straw biochar (300 °C) composite reached *q*_max_ of 247.9 mg/g. Corn stalk biochar-supported by nZVI even showed greater *q*_max_ towards Pb(II), which was 291.3 mg/g [8].

In recent years, nZVI has been increasingly added to SS prior to AD to enhance sludge biodegradability and improve CH_4_ production [25,26]. This addition increases the iron content of ADSS; consequently, biochar derived from such sludge is expected to contain elevated iron levels. However, to date, no studies have investigated how the addition of nZVI before AD affects the adsorption performance of biochar produced from the resulting ADSS. To the best of our knowledge, only one published study has investigated the effects of different pyrolysis temperatures and O_2_-exclusion methods (N_2_ purging and foil wrapping) on the physicochemical properties of sludge biochar [27], while the influence of these O_2_-exclusion methods on HM adsorption remains unclear. Therefore, this study employed biochar produced from nZVI-pretreated ADSS to evaluate the effects of pyrolysis temperature (300–800 °C) and O_2_-exclusion method (N_2_ purging versus foil-wrapping) on biochar yield and oxygen-containing functional groups. In addition, preliminary adsorption experiments were conducted to assess the effects of nZVI dosage applied prior to AD (5, 15, and 30 mg/g-TS) and pyrolysis atmosphere on the Pb(II) adsorption efficiency. Lead was selected as the target contaminant due to its significance as a major water pollutant [28]. To study the adsorption characteristics and mechanisms, various factors affecting adsorption of Pb(II) by the selected optimal biochar were evaluated, including adsorbent dosage, contact time, pH, coexisting ions, initial concentration of Pb(II), and temperature.

## 2. Materials and Methods

### 2.1. Materials

Thermally hydrolyzed sewage sludge (THSS) was used as the substrate for the AD and was collected from a municipal wastewater treatment plant (WWTP) located in Lithuania. Anaerobically digested sewage sludge from the same plant was used as an inoculum. Air-stable nZVI particles (NANOFER STAR) were purchased from Nano Iron s.r.o. (Židlochovice, Czech Republic). According to information from the supplier, the average particle size was 59.8  ±  1.3 nm, and SSA was 19.4 m^2^/g. The nZVI particles were activated by dispersing stable nZVI particles in deionized water at a ratio of 1:4.

A Pb(II) stock solution (1000 mg/L) was prepared by dissolving 1.62 g of lead nitrate (Pb(NO_3_)_2_) in 1 L of deionized water. Working solutions with concentrations ranging from 5 to 500 mg/L were then prepared by appropriate dilution of the stock solution. For the pH adsorption experiment, the solution pH was adjusted using 1 M HNO_3_ or NaOH [15]. All reagents were of analytical grade.

### 2.2. Adsorbent Preparation

The feedstock for biochar production was prepared by AD of sludge mixtures containing THSS (170 kg), an inoculum (60 kg), and different amounts of nZVI particles (0.065, 0.195, and 0.390 kg) in 300 L reactors. These nZVI amounts corresponded to dosages of 5, 15, and 30 mg per g of total solids (TS) of the sludge mixture [29]. A digester without nZVI was used as the control. AD was conducted for 40 days. Following digestion, the ADSS samples were dried at 105 °C to a constant weight and stored in sealed plastic bags for subsequent biochar production.

A preliminary study was carried out to examine whether the addition of nZVI before AD of thermally hydrolyzed SS influences the sorption properties of biochar produced from the resulting digestate (ADSSBC). For this purpose, digested non-pretreated (control) and nZVI-pretreated sludge samples were pyrolyzed in a muffle furnace (E5CC-T, SNOL, Utena, Lithuania) at 550 °C using two different O_2_-exclusion methods: (1) constant N_2_ (99.99%) gas flow (2 L/min), and (2) O_2_-limited conditions achieved by wrapping the samples in aluminum foil [21]. Then, in order to evaluate the effects of temperature and gas atmosphere on the properties of the biochar, the ADSS with optimal nZVI pre-treatment was slowly pyrolyzed at a fixed heating rate of 10 °C/min [3,8] and a wide temperature range from 300 °C to 800 °C (in 50 °C increments), using the same two O_2_-exclusion methods. Both the target temperature and heating rate were regulated by a built-in PID temperature controller. Prior to pyrolysis under a N_2_ atmosphere, the furnace was purged with N_2_ gas for 30 min. The samples were maintained at the target temperature for 2 h to ensure slow pyrolysis [30]. Approximately 134 g of digested sludge was pyrolyzed at each temperature and using each O_2_-exclusion method. After pyrolysis, the furnace was switched off, and the biochar was allowed to cool to room temperature (for pyrolysis under N_2_ atmosphere, cooling was carried out under continuous N_2_ flow). The produced biochar types were labeled according to the nZVI dosage and pyrolysis temperature used; for example, nZVIa-ADSSBC-550 refers to the biochar derived from nZVI pretreated ADSS at 550 °C, where “a” represents the nZVI dosage added to the sludge prior to AD. For the control sample, biochar derived from ADSS without the nZVI additive at 550 °C was labeled as ADSSBC-550. For subsequent experiments, the produced biochar was ground and sieved to obtain particles in the size range of 100–400 µm [31].

### 2.3. Characterization

Following pyrolysis, the biochar yield was assessed as an indicator of the mass efficiency of the pyrolysis process by evaluating the change in dry SS mass and biochar mass after pyrolysis [32]. The morphology and elemental composition of pristine biochar and Pb(II)-loaded biochar were analyzed using scanning electron microscopy coupled with energy dispersive spectroscopy (SEM-EDS) (FlexSEM 1000 II, Hitachi High-Tech Corporation, Hitachinaka, Japan; Oxford Instruments NanoAnalysis, High Wycombe, UK). The SSA (m^2^/g), total pore volume (TPV, cm^3^/g), and average pore diameter (nm) of the optimal biochar type were determined using a surface area and pore size analyzer (Sync 440A, 3P Instruments, Odelzhausen, Germany). The percentage pore size distribution relative to the total pore volume was calculated based on cumulative BJH desorption data. Prior to analysis, moisture and adsorbed gases were removed by outgassing the sample at 150 °C for 2 h under vacuum. Because surface functional groups are an important factor influencing the adsorption behavior of HMs, the spectral characteristics of biochar were analyzed by Fourier transform infrared spectroscopy (FTIR) in the 400–4000 cm^−1^ range (Invenio R, Bruker, Billerica, MA, USA) [8]. The original ADSS dried at 105 °C in an oven was used for comparison. X-ray diffraction (XRD) analysis of the optimal ADSSBC before and after Pb(II) adsorption was performed using an X-ray diffractometer (SmartLab SE, Rigaku, Tokyo, Japan). Data were collected over a 2θ range of 5–80° with a step size of 0.04° using Cu Kα radiation. X-ray photoelectron spectroscopy (XPS) analysis of the biochar before and after Pb(II) adsorption was carried out using a photoelectron spectrometer (Axis Supra Kratos Analytical Ltd., Manchester, UK) equipped with a monochromatic Al Kα source. The acquired spectra were analyzed using CasaXPS software (version 2.3.23rev1.1R). The point of zero charge (PZC), also known as the isoelectric point, of optimal biochar type was determined using the pH drift method [33,34]. PZC directly corresponds to the surface charge of biochar. Next, 0.1 g of the sample was weighed and mixed with 50 mL of 0.1 M NaCl solution, which was previously adjusted to initial pH (pH_i_) values from 1 to 12 by the addition of 0.1 M and/or 1 M HCl and/or NaOH solutions. The suspensions were agitated for 24 h using a rotary shaker (Rotoshake RS12, Gerhardt GmbH, Königswinter, Germany). After shaking, the pH of the solutions was recorded to determine the difference between the final and initial pH (ΔpH = pH_f_ − pH_i_). The intersection point of the resulting null ΔpH corresponds to PZC.

### 2.4. Adsorption Studies

In the adsorption experiments, Pb(II) was selected as the model contaminant due to its persistence and high toxicity [35]. To evaluate the influence of nZVI dosage applied prior to AD on the adsorption efficiency of ADSSBC, a preliminary adsorption experiment was conducted. A subsequent adsorption experiment was conducted to determine the optimal pyrolysis conditions for Pb(II) adsorption onto the ADSSBC. Finally, a third experiment was performed to examine the adsorption properties of the selected biochar by assessing the influence of key factors, including dosage, initial solution pH, coexisting ions, contact time, initial Pb(II) concentration, and temperature. All adsorption experiments (unless stated otherwise) were conducted in 100 mL glass bottles using 0.1 g of adsorbent and 50 mL of a 200 mg/L solution with a contact time of 360 min [5,7]. In the experiment on the effect of adsorbent dosage on adsorption capacity, the dosage was varied from 0.2 to 4.0 g/L. All samples (unless stated otherwise) were shaken at room temperature (22 ± 2 °C) and an agitation speed of 12 rpm using a rotary shaker. The effect of solution temperature on Pb(II) adsorption was investigated at 25, 35, 45, and 55 °C. The experiments were conducted in a shaking water bath (SW22, Julabo GmbH, Seelbach, Germany) at an agitation speed of 160 rpm and a contact time of 120 min [16,36]. All experiments (except the isotherm) were conducted at an initial pH of 5.0 ± 0.2 to avoid Pb(II) precipitation, with the solution pH left unadjusted prior to adsorption [37]. During the pH studies, solution pH values were adjusted between 1 and 5 using 1 M NaOH or HNO_3_ solutions prior to adsorbent addition [5,15,38]. For the kinetic experiment, samples were collected at predetermined time intervals from 5 min to 360 min. Since industrial wastewater typically contains other inorganic cations (e.g., K(I) and Ca(II)), which may compete for adsorption sites and thereby influence Pb(II) removal, these coexisting ions were included in the adsorption study to assess their effect on Pb(II) uptake by ADSSBC [37]. It was tested at a fixed Pb(II) concentration (200 mg/L) and varying Na(I) and Ca(II) concentrations (80–180 mg/L). A summary of all experimental conditions used in the adsorption studies is provided in Table 1.

After adsorption, the biochar was separated from the solution using 0.45 µm cellulose acetate syringe filters, and the filtrate was acidified with concentrated HNO_3_ prior to measurement. Residual Pb(II) concentration in the filtrate was determined using an inductively coupled plasma optical emission spectrometer (ICP-OES) (Avio 220 Max, Perkin Elmer, Waltham, MA, USA). The removal efficiency (*RE*), adsorption capacity at a given contact time (*q*_t_), and equilibrium adsorption capacity (*q*_e_) for Pb(II) were calculated using the following equations [39]:

(1) $$RE=\frac{C_{0}-C_{e}}{C_{0}}\times 100\%,$$

(2) $$q_{t}=\frac{(C_{0}-C_{t})\times v}{m},$$

(3) $$q_{e}=\frac{(C_{0}-C_{e})\times v}{m},$$

where, *C*_0_, *C*_t_, and *C*_e_ denote the initial, time-specific, and equilibrium concentrations of Pb(II) in the solution (mg/L), respectively; *v* represents the volume of the solution (L), and *m* is the adsorbent mass (g). To elucidate the mechanism of Pb(II) removal, lead-containing biochar was dried at 105 °C and analyzed using FTIR, XRD, XPS, and SEM-EDS [15].

### 2.5. Kinetic, Isotherm, and Thermodynamic Models

Pb(II) adsorption mechanism was assessed using three non-linear kinetic models and two non-linear isotherm models. The kinetic data were fitted to the pseudo-first-order (PFO), pseudo-second-order (PSO), and intra-particle diffusion (IPD) models, while the adsorption isotherms were analyzed using the Langmuir and Freundlich models. All curve fitting analyses were carried out with Origin software (version 2019b, OriginLab Corporation, Northampton, MA, USA). The non-linear forms of the kinetic and isotherm models used in this study are presented in Table 2. PFO model characterizes monolayer adsorption, which is dominated by boundary layer diffusion processes [40]. The PSO model assumes that the adsorption rate is governed by a chemisorption mechanism in which electron sharing or transfer occurs between the adsorbent and adsorbate. The IPD model can be used to assess whether the overall sorption process is controlled by the adsorption rate. To evaluate the suitability of the optimal biochar for Pb(II) adsorption, the dimensionless separation factor, *R*_L_, was determined using the Langmuir isotherm model [41]. According to the *R*_L_, the adsorption process is considered unfavorable when *R*_L_ > 1, linear when *R*_L_ = 1, favorable when 0 < *R*_L_ < 1, and irreversible when *R*_L_ = 0 [42,43]. Thermodynamic parameters, including Gibbs’ free energy change (Δ*G*^0^), enthalpy change (Δ*H*^0^), and entropy change (Δ*S*^0^), were calculated using the equations shown in Table 2. The Gibbs free energy change serves as a fundamental criterion for assessing adsorption spontaneity, the enthalpy change indicates whether the process is endothermic or exothermic, and the entropy change reflects the degree of disorder within the system.

### 2.6. Statistical Analysis

All physicochemical analyses were performed in triplicate. Statistical analyses (means ± standard deviations, SD) and graph preparation were carried out using Microsoft Office Excel (2021). All adsorption experiments were conducted in duplicate, and extra analyses were carried out if the two measurements differed by more than 5% [48]. A two-way ANOVA was employed to evaluate the effects of pyrolysis temperature and O_2_-exclusion method and their interaction on the biochar yield, as well as its Pb(II) adsorption capacity, using IBM SPSS Statistics software (version 30.0; IBM Corp., Armonk, NY, USA). When a significant effect was detected, Tukey’s honestly significant difference (HSD) post hoc test was applied to determine pairwise differences between treatment means at the 95% confidence level (*p* < 0.05). The coefficient of determination (*R*^2^) was used to assess the goodness of fit between the experimental and predicted data for adsorption kinetics and isotherms [49]. Some studies indicate that using only *R*^2^ may not adequately represent model fit. Thus, the root mean squared error (*RMSE*) and chi-square (*χ*^2^) were included to evaluate the model fit, as small values of these parameters indicate that the model predictions closely match the experimental results [50,51].

## 3. Results and Discussion

### 3.1. Effect of Pyrolysis Temperature and O_2_-Exclusion Method on Biochar Yield

**Figure 1:**
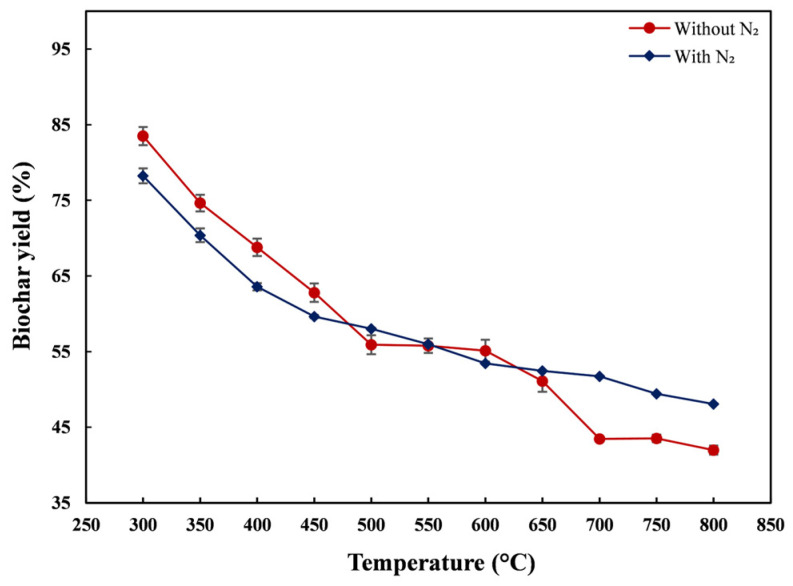
Influence of pyrolysis temperature and O_2_-exclusion method on the yield of biochar produced from nZVI30-ADSS. Data are presented as mean ± SD (*n* = 2).

Biochar yield results showed that nZVI30-ADSSBC yield decreased with increasing pyrolysis temperature under both O_2_-exclusion conditions (Figure 1). From 300 to 800 °C, the yield decreased by approximately 30 and 42 percentage points under N_2_ and foil-wrapped conditions, respectively, indicating that the N_2_ atmosphere provided more effective O_2_ exclusion and reduced carbon loss during pyrolysis. The decrease in biochar yield may be associated with continued pyrolysis conversion, attributable either to more extensive primary decomposition of biomass or to secondary reactions of the remaining solid residue [52]. At temperatures above 500 °C, the yield of biochar obtained under the N_2_ atmosphere was slightly higher than that obtained under foil-wrapped conditions. The higher biochar yield under N_2_ can be attributed to the more efficient removal of O_2_, which minimized oxidation and secondary decomposition of nZVI30-ADSSBC during pyrolysis. In contrast, the foil-wrapping method likely allowed limited O_2_ diffusion, enhancing volatile losses and reducing yield. Two-way ANOVA indicated that biochar yield was significantly influenced by pyrolysis temperature (*p* < 0.001), whereas the O_2_-exclusion method and the interaction between temperature and O_2_-exclusion method showed no significant effects (*p* > 0.05). Tukey’s post hoc test revealed that the nZVI30-ADSSBC yield decreased significantly with increasing pyrolysis temperature, with yields at 700–800 °C being significantly lower than those at 300–500 °C (*p* < 0.05). These results are similar to those obtained by Yuan et al. [53], who reported that the yield of biochar produced from digested sludge decreased from 83.3% to 65.0% as the pyrolysis temperature increased from 300 °C to 700 °C under a N_2_ atmosphere. In this study, the lowest biochar yields were obtained at 700–800 °C under foil-wrapped conditions, reaching 42.0–43.5%. These SS-derived biochar yields are considerably higher than those of other biomass types reported in the literature, including pine wood (14.2% at 700 °C) and hemp (11.2% at 700 °C) [54]. This relatively high yield is commonly attributed to the substantial mineral fraction in SS, such as phosphorus, silica, and various metal compounds that are retained in the solid phase during pyrolysis. Similarly, Tang et al. [55] showed that metal salt-impregnated biomass results in a higher biochar yield compared to pristine biomass.

### 3.2. Effect of Pyrolysis Temperature and O_2_-Exclusion Method on Biochar Surface Chemistry

**Figure 2:**
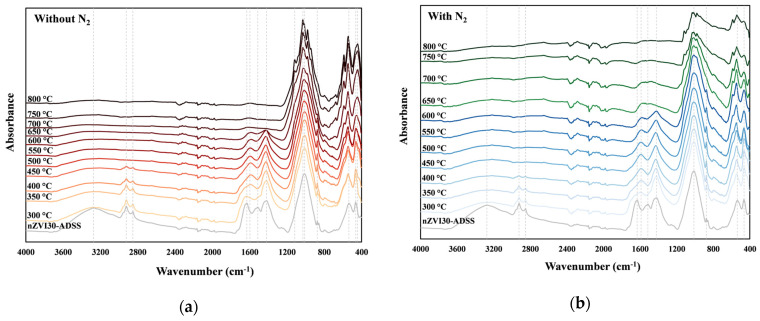
Comparison of temperature-dependent FTIR spectra of nZVI30-ADSS and its derived biochar produced (**a**) without N_2_ and (**b**) with N_2_. The vertical dashed lines indicate the characteristic absorption bands discussed in the text.

Figure 2 presents the FTIR spectra of the raw nZVI30-ADSS sample and the corresponding biochar types produced at 300–800 °C under two different conditions: foil-wrapped (Figure 2a) and in a N_2_ atmosphere (Figure 2b). In the raw nZVI30-ADSS sample, a broad absorption band around 3275 cm^−1^ is assigned to –OH and –NH stretching vibrations originating from the organic matter present in SS [56]. However, it can be observed that under both atmospheric conditions, the intensity of this peak drastically decreased when the samples were heated to 300 °C and higher temperatures, indicating the instability of –OH and –NH surface functional groups. Absorption peaks observed at 2924 cm^−1^ and 2852 cm^−1^ correspond to C–H stretching vibrations, confirming the presence of aliphatic components in ADSS. These bands disappeared at 500 °C in the absence of N_2_ and at 450 °C in its presence. A similar observation for SS biochar was reported by Zhang et al. [57], where the C–H peak disappeared at 500 °C, indicating the breakdown of aliphatic compounds into gaseous products. These functional groups are thermally unstable and decompose at relatively low temperatures, thereby contributing to increased mass loss and enhanced gas evolution during thermal decomposition [56]. As the pyrolysis temperature increased, the characteristic –CO–NH– peak shifted from 1631 cm^−1^ to an average of 1597 cm^−1^ under N_2_-free conditions and to an average of 1588 cm^−1^ in the presence of N_2_. This may have been caused by complexation between acylamino groups and HMs during pyrolysis [57]. It can be observed that, in the absence of an N_2_, the peak at 1597 cm^−1^ completely disappeared at 700 °C, whereas under an N_2_ atmosphere this peak remained slightly more pronounced. This indicates that even at elevated temperatures, biochar produced under N_2_ better preserves N-containing functional groups. In another study, one of the dominant functional groups identified in graphene oxide (GO)–modified biochar derived from ADSS was assigned to the C=N group (1599 cm^−1^), whose peak intensity was stronger compared to that of GO-modified rice husk biochar [58]. In raw ADSS pretreated with 30 mg/g-TS nZVI, the band at 1514 cm^−1^ corresponds to –NH vibrations [59], which were eliminated during the pyrolysis process. The peak at 1419 cm^−1^ was attributed to –CH_2_ and –CH_3_ vibrations and disappeared at 700 °C without N_2_ atmosphere (Figure 2a) and at 650 °C in its presence (Figure 2b). A similar observation was reported by Zhang et al. [57], who observed that fatty chain groups (peak between 1410 cm^−1^ and 1430 cm^−1^) only disappeared at 700 °C for SS under N_2_ atmosphere due to the high stability of C–H bonds [60]. All samples exhibited a strong band at 1011 cm^−1^, which shifted to 1030 cm^−1^ for biochar produced at ≥700 °C under N_2_-free conditions, indicating the presence of a stable C–O bond. The absorption peak observed at 1030 cm^−1^ corresponds to the C–C and C–O bond characteristics of the ester functional groups [59]. The peak at around 876 cm^−1^ was attributed to a highly stable C–H stretching vibration [61], which vanished only at 700 °C under N_2_-free conditions and at 650 °C in the presence of N_2_. Absorption peaks below 600 cm^−1^ indicate the presence of inorganic compounds [56].

### 3.3. Effect of nZVI Dosage, Pyrolysis Temperature, and O_2_-Exclusion Method on Pb Removal Performance by Biochar

**Figure 3:**
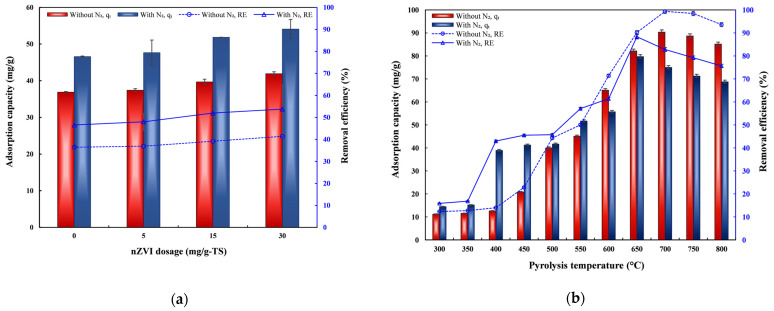
Effects of (**a**) nZVI dosage applied before AD and the O_2_-exclusion method, and (**b**) pyrolysis temperature and O_2_-exclusion method on the lead removal performance of nZVI30-ADSSBC. Experimental conditions: biochar dosage = 2 g/L, initial pH = 4.9, *t* = 360 min, *C*_0_ = 200 mg/L, and *T* = 20–22 °C. Data are presented as mean ± SD (*n* = 2).

The Pb(II) *q*_t_ and *RE* varied with nZVI dosage and the O_2_-exclusion method (Figure 3a). At 550 °C, the ADSSBC prepared under N_2_ exhibited higher *q*_t_ and *RE* than biochar prepared under foil-wrapped conditions. Biochars prepared under N_2_ consistently exhibited higher Pb(II) adsorption capacities (46.6–54.1 mg/g) compared to those prepared under foil-wrapped conditions (36.9–41.9 mg/g), and the effect of atmosphere was found to be strong and statistically significant (*p* < 0.001). These results indicate that the N_2_ atmosphere better preserved the reactivity of biochar at 550 °C, enhancing Pb(II) adsorption. Since the highest *q*_t_ was obtained for ADSSBC derived from 30 mg/g-TS nZVI-pretreated sludge, this ADSSBC was selected for further studies on Pb(II) removal across different pyrolysis temperatures and atmospheres (Figure 3b). Because *q*_t_ is positively correlated with *RE*, both curves followed an identical trend. For both atmospheres, the adsorption performance increased with increasing temperature. However, the temperature at which the maximum *q*_t_ was achieved differed between the two treatments. For biochar produced without N_2_, *q*_t_ gradually increased from 300 °C to 700 °C, reaching a maximum adsorption capacity of 90.4 mg/g at 700 °C, and then slightly decreased at higher temperatures (750–800 °C). The corresponding *RE* reached 99.4% at 700 °C. For the biochar produced with N_2_, the maximum *q*_t_ (79.8 mg/g) was obtained at 650 °C, whereas a decline in *q*_t_ was observed at 700–800 °C. Overall, the results indicate that, independent of the atmosphere used, *q*_t_ increased with increasing pyrolysis temperature until reaching an optimum value at high temperatures, which may be explained by the possibly enhanced SSA and TPV of the biochar produced at elevated temperatures. Multiple studies have reported that the SSA and TPV of ADSSBC increase as the pyrolysis temperature increases [53].

The Pb(II) *RE* of nZVI30-ADSSBC prepared under N_2_ increased steadily to about 88.3% at 650 °C. At higher temperatures (600–800 °C), it was consistently lower than that of the biochar produced without N_2_. Pb(II) adsorption capacity increased the most from 350 to 400 °C, when *q*_t_ increased by 2.56 times, and from 600 to 650 °C, when *q*_t_ increased by 1.43 times. In general, the results showed that pyrolysis without N_2_ led to higher adsorption capacity and *RE* at elevated temperatures, suggesting that the absence of N_2_ may promote the formation of a more porous structure or surface chemistry that is favorable for adsorption. Thus, nZVI30-ADSSBC, produced at 700 °C without N_2_, was identified as the optimal biochar and was used in subsequent adsorption experiments. The literature review showed that biochar produced under an N_2_ atmosphere at a pyrolysis temperature of 650 °C from various other feedstocks also exhibited the highest lead removal capacity when compared with biochars produced at other pyrolysis temperatures. For example, a study conducted by Wang et al. [40] found that biochar produced at 650 °C showed the highest lead adsorption capacity (98.9 mg/g) when using *Caragana korshinskii* biomass under N_2_ conditions. He et al. [62] evaluated the effect of pyrolysis temperature (350–750 °C) on Pb(II) removal using magnetite-modified coffee-ground biochar and found that the biochar produced at 650 °C under an N_2_ atmosphere exhibited the highest Pb(II) *RE* (approximately 55%).

### 3.4. Effect of Biochar Dosage on Pb(II) Removal Performance

**Figure 4:**
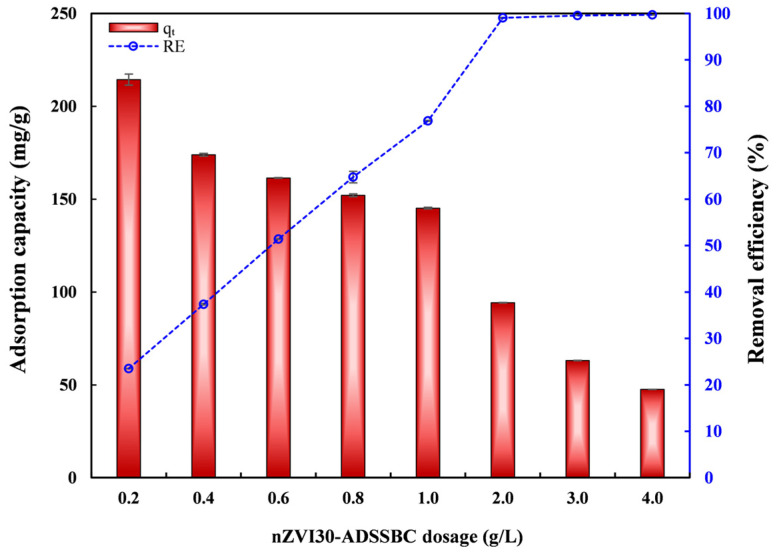
Effect of nZVI30-ADSSBC-700 dosage on lead removal performance. Experimental conditions: initial pH = 4.9, *t* = 360 min, *C*_0_ = 200 mg/L, and *T* = 23 °C. Data are presented as mean ± SD (*n* = 2).

Adsorbent dosage plays a crucial role in adsorption performance, as it influences both the adsorption capacity rate and *RE*. The impact of adsorbent dosage on Pb(II) *q*_t_ and *RE* is shown in Figure 4. Pb(II) *RE* increased steadily from 23.5% to 99.1% as the adsorbent dosage increased from 0.2 to 2.0 g/L, due to the greater number of available active sites, higher SSA, and more functional groups at higher doses, which enabled increased Pb(II) uptake. Above 2.0 g/L, the increase in *RE* became negligible up to 4.0 g/L, likely because particle aggregation and active site overlap reduced the effective SSA and adsorption efficiency per unit mass. Thus, a dosage of 2.0 g/L was determined to be optimal for achieving maximum Pb(II) removal and was selected for subsequent experiments, providing a practical balance between *RE* and cost. It can also be seen that the adsorption capacity decreased at higher dosages, probably due to inefficient site utilization and particle aggregation. Comparable results were reported in the literature, showing that increasing the dosage of sludge-based adsorbents enhances removal efficiency for Pb(II) [5].

### 3.5. Effect of Initial pH on Pb(II) Removal Performance

**Figure 5:**
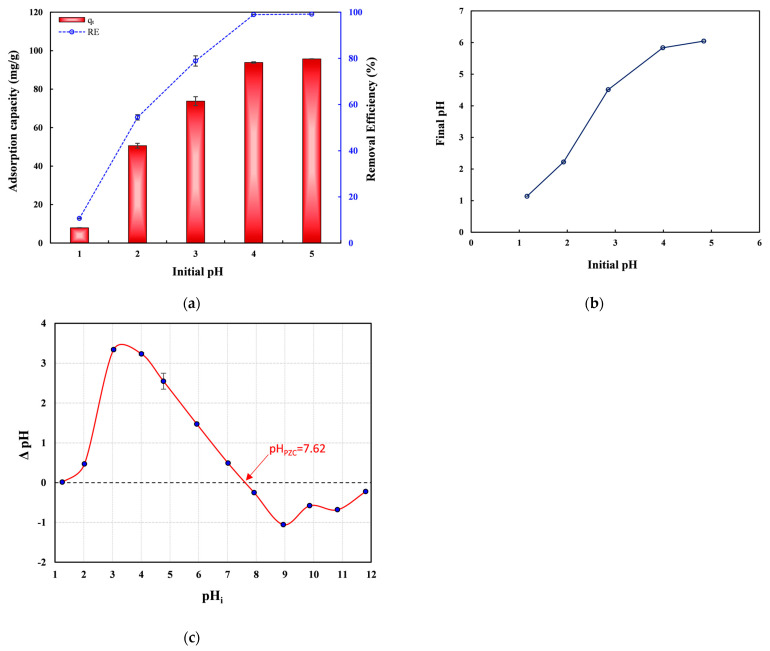
(**a**) Effect of initial solution pH on Pb(II) adsorption capacity and removal efficiency by nZVI30-ADSSBC-700; (**b**) pH changes before and after Pb(II) adsorption; (**c**) pH point of zero charge of nZVI30-ADSSBC-700 determined by the pH drift method. Experimental conditions: biochar dosage = 2 g/L, *t* = 360 min, *C*_0_ = 200 mg/L, and *T* = 23 °C. Data are presented as mean ± SD (*n* = 2).

Because solution pH strongly influences adsorbent surface charge and the chemical forms of Pb(II), the adsorption performance of nZVI30-ADSSBC-700 was evaluated at initial pH values ranging from 1.0 to 5.0. Since Pb(II) exists predominantly as a free cation below pH 6 [45], the experimental pH values were chosen to be ≤5 to avoid precipitation of Pb as hydroxides at higher pH values and to ensure valid adsorption measurements and data interpretation [46]. As the pH increased from 1 to 4, Pb(II) removal increased correspondingly but remained stable at pH values above 4 (Figure 5a). The adsorption capacity was almost the same in the pH range of 4–5. This is consistent with another study that reported that the Pb(II) adsorption capacity increased as pH increased from 2 to 4 and then remained stable between pH 4 and 6 when ZVI-containing magnetic biochar derived from waste-activated sludge was used [63]. It can be seen that the lowest Pb(II) adsorption capacity and *RE* (7.97 mg/g and 10.7%) occurred under strongly acidic conditions (pH = 1). This low adsorption was caused by competition between the cations released from the biochar (K(I), Na(I), Ca(II), and Mg(II)) with Pb(II) for the active sites, as well as an increase in the positive charge density of the surface of biochar [5]. Additionally, at low pH, the high concentration of H_3_O^+^ ions competes with Pb(II) for adsorption sites, leading to reduced Pb(II) uptake [43]. Other studies have also demonstrated that an increased concentration of H^+^ leads to lower Pb(II) adsorption [64]. At pH 5, *q*_t_ and *RE* reached their peaks (95.7 mg/g and 99.2%, respectively). As the pH increases, the competitive adsorption between Pb(II) and H_3_O^+^ ions becomes progressively weaker; therefore, *q*_t_ increases as the pH increases [41]. Based on these results, pH 5 was chosen as the optimal condition for subsequent experiments. Additionally, since the pH of real wastewater containing different metal ions is also around 5, this can help reduce operational costs associated with pH adjustment [62]. The final pH of the solution was measured after adsorption (Figure 5b). The point of zero charge (PZC) indicates the pH at which the adsorbent surface is neutral. The pH_PZC_ of nZVI30-ADSSBC-700 was estimated from the plot of the pH difference (between pH_f_ and pH_i_) against pH_i_ (Figure 5c). The pH_PZC_ of the produced adsorbent was 7.6, indicating that the surface of the adsorbent would be positive at solution pH values up to pH 7.6, after which it would become negative. As both Pb(II) ions and biochar surfaces are positively charged, there is no electrostatic interaction between them until a pH of 7.6, and the chance of electrostatic attraction appears to be at solution pH above the aforementioned PZC. These findings are similar to those of Shao et al. [7], who reported a pH_PZC_ value of 7.5 for municipal sludge biochar.

### 3.6. Effect of Coexisting Ions

**Figure 6:**
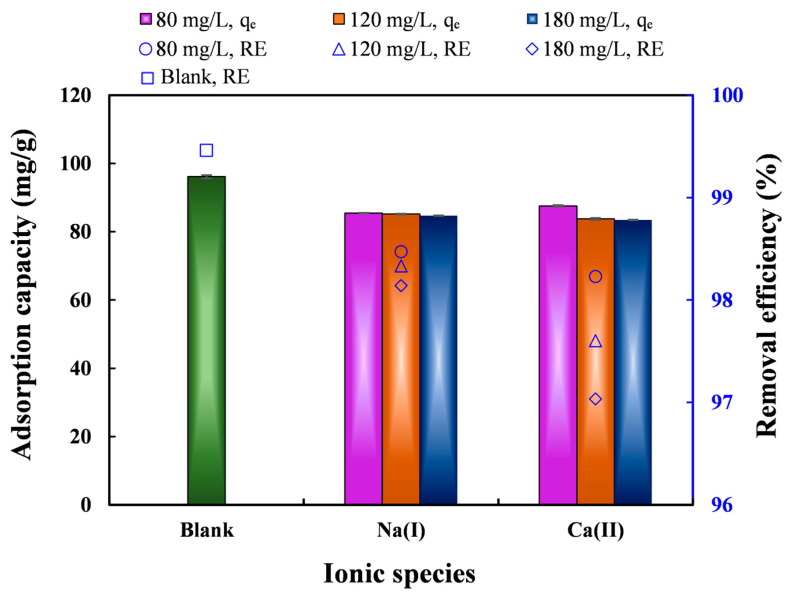
Effect of competing ionic species on Pb(II) adsorption capacity and removal efficiency. Experimental conditions: biochar dosage = 2 g/L, initial pH = 4.9, *t* = 360 min, *C*_0_ = 200 mg/L, and *T* = 24 °C. Data are presented as mean ± SD (*n* = 2).

It is important to examine how coexisting ions present in water affect Pb(II) adsorption on biochar. The selectivity of the adsorbent towards Pb(II) was assessed by examining the effect of different Na(I) and Ca(II) concentrations on Pb(II) *q*_t_ and *RE*. Elevated levels of Na(I) and Ca(II) increased their competitive interaction with Pb(II) for sorption sites on nZVI30-ADSSBC-700 (Figure 6). The co-existence of Ca(II) reduced the Pb(II) *q*_t_ more strongly at higher initial ion concentrations (120–180 mg/L) compared to Na(I). Pb(II) *q*_t_ decreased in the presence of Na(I) by 11.41% and 11.91% at 120 and 180 mg/L, respectively, and in the presence of Ca(II) by 12.87% and 13.19% at 120 and 180 mg/L, respectively. In comparison with the blank (only Pb(II)), the presence of Na(I) and Ca(II) did not significantly affect Pb(II) *RE*, since it remained above 97% even at a competing cation concentration of 180 mg/L. The stronger inhibitory effect of Ca compared to that of Na can be attributed to its higher charge and larger hydrated ionic radius, which enhances its electrostatic interaction with negatively charged adsorption sites and increases competition with Pb(II). According to the Pauling scale, the electronegativities of Pb, Ca, and Na are 1.87, 1.00, and 0.93, respectively, while their hydrated ionic radii are 0.401 nm, 0.412 nm, and 0.358 nm [65,66,67]. A study conducted by Xu et al. [68] similarly found that Ca(II) had a stronger inhibitory effect on Pb(II) removal by magnetic coconut biochar than comparable concentrations of Na(I) and K(I), due to the impact of Ca(II) on limiting Pb(II) precipitation on the adsorbent surface.

### 3.7. Adsorption Kinetics

**Figure 7:**
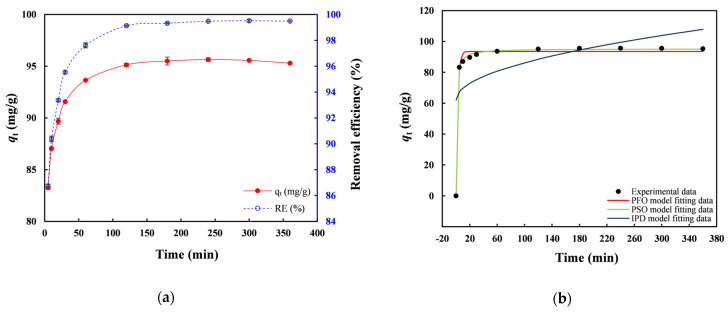
(**a**) Effect of contact time on Pb(II) adsorption by nZVI30-ADSSBC-700 at an initial Pb(II) concentration of 200 mg/L; (**b**) Adsorption kinetics of nZVI30-ADSSBC-700. Experimental conditions: biochar dosage = 2 g/L, initial pH = 4.9, *C*_0_ = 200 mg/L, and *T* = 22 °C. Data are presented as mean ± SD (*n* = 2).

Figure 7 presents the kinetic results of Pb(II) sorption by nZVI30-ADSSBC-700 obtained using contact times between 5 and 360 min with an initial Pb(II) concentration of 200 mg/L. All experimental variables were controlled, with contact time being the only one that was varied. Adsorption capacity and *RE* increased exponentially during the first 120 min due to interactions between Pb(II) ions and abundant free binding sites on biochar, reaching equilibrium within this time interval (*q*_e_ = 95.1 mg/g and *RE* = 99.1%) (Figure 7a). Adsorption then remained stable over the next 240 min as the sites became saturated. Notably, 87.0% of the equilibrium adsorption capacity was achieved within 5 min and more than 99% within 120 min. The initial rapid uptake was followed by a slower adsorption stage, which was likely controlled by ion-exchange processes [40].

Dynamic adsorption data for Pb(II) uptake on nZVI30-ADSSBC-700 were fitted using the PFO, PSO, and IPD models (Figure 7b). The parameters obtained from the kinetic models are presented in Table 3. It can be seen that both the PFO and PSO models fit the data very well, as indicated by their similarly high *R*^2^ values (>0.999). However, the PSO model showed a slightly higher *R*^2^ and lower error function values, indicating a marginally better fit than the PFO model. The PSO kinetic model showed the highest accuracy among the three models, providing the best fit to the experimental data, with the highest *R*^2^ value (0.9999), the lowest *RMSE* (0.97), and the lowest *χ*^2^ value (0.10). This model also provided the calculated *q*_e_ value (*q*_e_ = 95.21 mg/g), which was closest to the experimental value (*q*_e,exp_ = 95.13 mg/g) compared with all the kinetic models studied. This suggests that Pb(II) adsorption was primarily driven by chemisorption, although physisorption also contributed to the overall adsorption process. This is consistent with the findings of Ho et al. [15], who also reported that the PSO model provided the best fit (*R*^2^ = 0.97) to the dynamic experimental data for lead sorption at an initial concentration of 200 mg/L using biochar (600 °C) derived from AD sludge. Wang et al. [5] similarly showed that the PSO model provided a better fit to the experimental data than the PFO model in the case of biochar derived from SS and calcium sulfate. This indicates that chemisorption governs Pb(II) removal by different sludge-based biochar adsorbents.

### 3.8. Adsorption Isotherms

**Figure 8:**
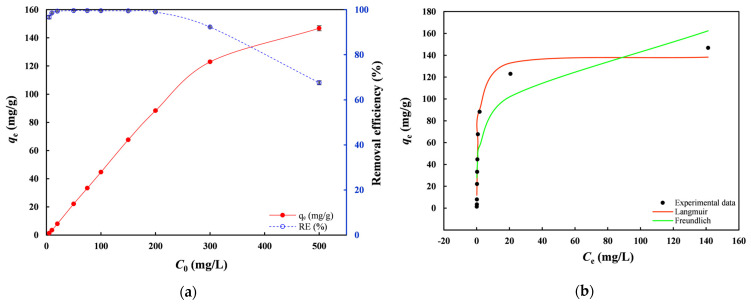
(**a**) Effect of initial Pb(II) concentration on Pb(II) adsorption capacity and removal efficiency; (**b**) Adsorption isotherm fit curves for experimental Pb(II) adsorption data. Experimental conditions: biochar dosage = 2 g/L, *t* = 120 min, initial pH is natural (4.8–5.4). Data are presented as mean ± SD (*n* = 2).

Figure 8a shows that *q*_e_ and *RE* followed distinct trends as the initial concentration (*C*_0_) increased. It can be seen that nZVI30-ADSSBC-700 was very efficient for Pb(II) removal below 200 mg/L, with an *RE* above 99%. However, the *RE* of Pb(II) decreased from 98.9% to 67.6% as *C*_0_ increased from 200 mg/L to 500 mg/L. However, over a wide range of *C*_0_ (5–200 mg/L), *RE* was sufficiently stable, ranging from 96.7% at 5 mg/L to 99.6% at 50–75 mg/L. Therefore, it can be claimed that nZVI30-ADSSBC-700 did not become saturated under the mentioned conditions, as *RE* was very close to 100%. In contrast, *q*_e_ tended to increase with increasing initial Pb(II) concentration because the enhanced driving force minimized the mass transfer resistance between the solution and biochar.

The Langmuir and Freundlich models were applied to describe Pb(II) adsorption on nZVI30-ADSSBC-700 (Figure 8b), with the fitted isotherm parameters listed in Table 4. The results showed that the Langmuir model (*R*^2^ = 0.99) better described the experimental data compared to the Freundlich model (*R*^2^ = 0.92), indicating that Pb(II) adsorption by nZVI30-ADSSBC-700 was monolayer chemical adsorption, i.e., the entire surface had identical adsorption capacity due to the uniform distribution of active sites on the adsorbent surface [7,15]. The *q*_max_ obtained from the Langmuir model was 139.3 mg/g, which was slightly lower compared to the experimental value (146.8 mg/g). According to the obtained 1/*n* value (0.24) from the Freundlich model, the adsorption process can be ascribed as favorable due to a value between 0 and 1 [7,47]. This is also consistent with the calculated *R*_L_ values, which decreased from 0.164 to 0.002 as the initial Pb(II) concentration increased from 5 to 500 mg/L, indicating highly favorable adsorption over the entire concentration range. Similar fitting results, when the Langmuir model outperformed other models, have been reported for Pb(II) adsorption onto sludge biochar derived from cow manure and its vermicompost [38], nZVI/biochar composite [46], pharmaceutical sludge-based biochar [7], and magnetic biochar derived from persulfate-ZVI treated sludge [63].

### 3.9. Thermodynamic Analysis

**Figure 9:**
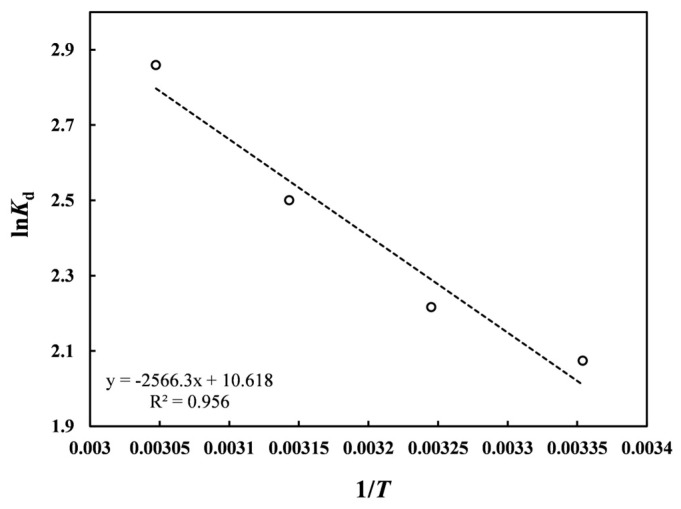
Plot of ln*K*_d_ versus 1/*T* for Pb(II) adsorption by nZVI30-ADSSBC-700.

To investigate the effect of temperature on Pb(II) adsorption on nZVI30-ADSSBC-700, adsorption thermodynamics at different temperatures (298–328 K) were analyzed. The high *R*^2^ value (0.96) indicates a strong agreement between the experimental adsorption data and the Van’t Hoff model, confirming the reliability of the calculated thermodynamic parameters (Figure 9). The slope (−Δ*H*^0^/*R*) and intercept (Δ*S*^0^/*R*) obtained from the Van’t Hoff plot were used to determine the thermodynamic parameters (Δ*G*^0^, Δ*H*^0^ and Δ*S*^0^), which are presented in Table 5. Negative Δ*G*^0^ values (from −4.98 kJ/mol to −7.63 kJ/mol in the temperature range of 298–328 K) confirmed that Pb(II) adsorption on the adsorbent was spontaneous and became more favorable at higher temperatures because of the increased driving force [5]. This suggests that the system exhibits a greater degree of spontaneity with increasing temperature, mainly owing to chemisorption rather than physisorption. This indicates that a relatively high temperature (328 K) is advantageous for Pb(II) adsorption on nZVI30-ADSSBC-700. The increase in temperature provided more energy, making it easier for the Pb(II) ions to adsorb onto the biochar surface. Such adsorption following an endothermic path has been reported for other biochar adsorbents [47,51,64]. The adsorption capacity increased with temperature, and the positive Δ*H*^0^ value (21.34 kJ/mol) confirmed that the adsorption process was endothermic and physico-chemical, as this value was between 21 and 80 kJ/mol [69]. The positive value of Δ*S*^0^ (88.28 J/(mol∙K)) indicates an increase in disorder and randomness at the solid-solution interface during adsorption, probably due to the release of ions from the nZVI30-ADSSBC-700 surface into the solution [64]. Similarly, Wei et al. [70] have reported Δ*G*^0^, Δ*H*^0,^ and Δ*S*^0^ values of from −3.28 kJ/mol to −6.72 kJ/mol (at 293.15–333.15 K), 21.9 kJ/mol, and 86.4 J/(mol∙K), respectively, for the adsorption of Pb(II) onto rice husk biochar-supported nZVI composite.

### 3.10. Comparison with Other Adsorbents

The *q*_max_ of nZVI30-ADSSBC-700 was compared with the capacities reported for other similar adsorbent materials (Table 6). The *q*_max_ of the adsorbent reached 139.3 mg/g according to the Langmuir model. This value exceeded those of most previously reported sludge-based adsorbents, demonstrating the strong potential of this biochar for the removal of Pb(II) from contaminated water.

**Figure 10:**
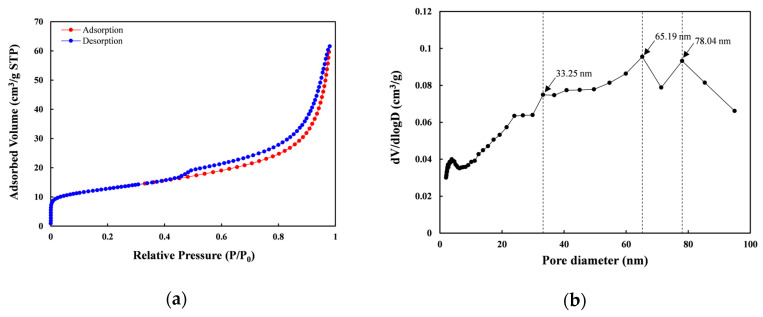
(**a**) N_2_ adsorption/desorption isotherms; (**b**) Pore size distribution of nZVI30-ADSSBC-700 according to Barrett–Joyner–Halendar (BJH) theory.

According to the IUPAC classification [71], the adsorption-desorption isotherms of nZVI30-ADSSBC-700 were identified as Type IV based on their physisorption profile, exhibiting an H3-type hysteresis loop (Figure 10a). This confirms that the adsorbent is predominantly mesoporous and consists of plate-like particles and slit-shaped pores within the biochar structure. According to the pore size distribution graph (Figure 10b), nZVI30-ADSSBC-700 exhibits a hierarchical structure containing both mesopores (2–50 nm) and macropores (>50 nm), with a predominant presence of the former. In terms of total pore volume, mesopores accounted for 87.8%, macropores for 11.7%, and micropores for 0.48%. A pore structure formed by joining meso- and macropores establishes effective transport pathways that enhance the distribution of pollutant molecules throughout the biochar [72,73]. The resulting biochar exhibited an SSA of 45.7 m^2^/g, TPV of 0.095 cm^3^/g, and an average pore diameter of 8.35 nm (mesopores). Compared to other sewage sludge-based sorbents (Table 6), the SSA of the biochar obtained in this study was the highest, indicating that the surface area played a significant role in lead sorption. Conversely, despite having a smaller SSA, SS-and calcium sulfate-derived biochar [5] exhibited a slightly higher Pb uptake than that observed in our study. This performance was likely driven by mechanisms beyond surface area, specifically mineral precipitation (PbSiO_3_), ion exchange between Pb(II) and Ca(II), and the presence of oxygen-containing functional groups (–OH and –COOH). These groups, modified following CaSO_4_ impregnation of the SS, facilitated Pb(II) removal through enhanced chemical interactions.

Comparing the results of this study with those of Chen et al. [63], who demonstrated a 33% higher Pb(II) sorption capacity on magnetic biochar produced from persulfate-ZVI dewatered waste activated sludge (139.3 mg/g and 206.5 mg/g, respectively), it can be hypothesized that the superior performance in the aforementioned study was likely due to a 67-fold higher loading of ZVI compared to that in the present study. In this study, the nZVI dosage was limited to 30 mg/g TS because higher dosages can inhibit methanogenesis, as identified in our previous study on SS AD with nZVI [29]. Therefore, applying nZVI after AD, rather than before, could allow the use of higher nZVI loadings without negatively affecting AD performance. Consequently, to produce a more efficient sorbent from ADSSBC relative to the results of the cited study, it is recommended to obtain ADSS directly from WWTPs and incorporate a higher amount of nZVI particles prior to SS pyrolysis.

### 3.11. Mechanisms for Pb(II) Removal by nZVI30-ADSSBC-700

#### 3.11.1. Precipitation

**Figure 11:**
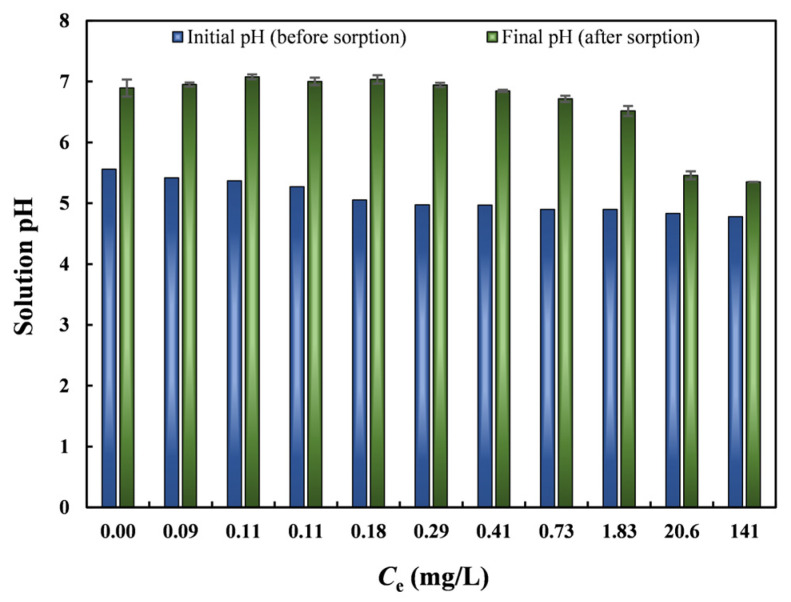
Changes in solution pH during the Pb(II) adsorption isotherm experiment. Experimental conditions: biochar dosage = 2 g/L; *t* = 120 min. Data are mean ± SD (*n* = 2).

The observed differences between the initial and final solution pH values at varying Pb(II) concentrations in the isotherm experiment support surface precipitation as a mechanism of Pb(II) removal by the studied biochar. At low equilibrium Pb(II) levels (0.00–1.83 mg/L), the pH increased from 5.7 to 6.9 on average, reflecting the alkalinity of the biochar and the release of carbonates into the solution (Figure 11). The results show that as the equilibrium Pb(II) concentration increased further from 1.83 mg/L to 141 mg/L, the pH value decreased to ~5.5 due to the complete consumption of available alkali ions. The observation that the final pH reached its lowest value at the same solvent concentration where the isotherm curve leveled off further supports the significance of the surface precipitation mechanism. This is consistent with the findings of Inyang et al. [44], who reported that the pH reached its minimum at the solute concentration where the Pb(II) isotherm attained a plateau, indicating the initiation of a surface-precipitation mechanism.

**Figure 12:**
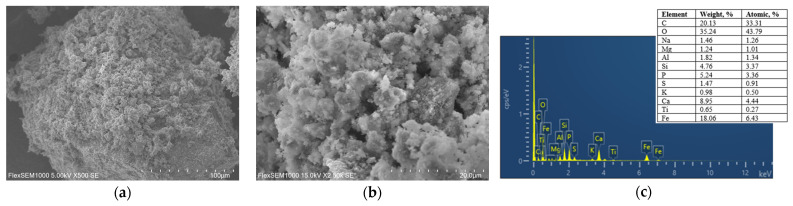

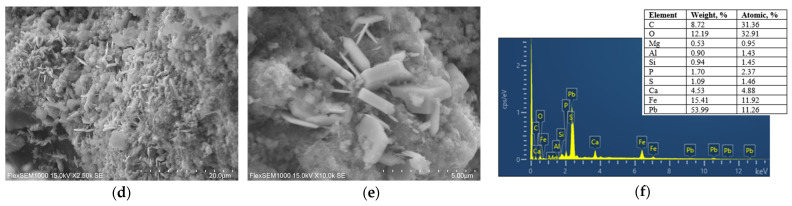
(**a**,**b**) SEM images of nZVI30-ADSSBC-700 before Pb(II) adsorption at 500× and 2500× magnifications; (**c**) EDS spectrum of the biochar particle before Pb(II) adsorption; (**d**,**e**) SEM images of Pb-loaded nZVI30-ADSSBC-700 at 2500× and at 10,000× magnifications; (**f**) EDS spectrum corresponding to the area shown in (**e**) for Pb-loaded nZVI30-ADSSBC-700.

**Figure 13:**
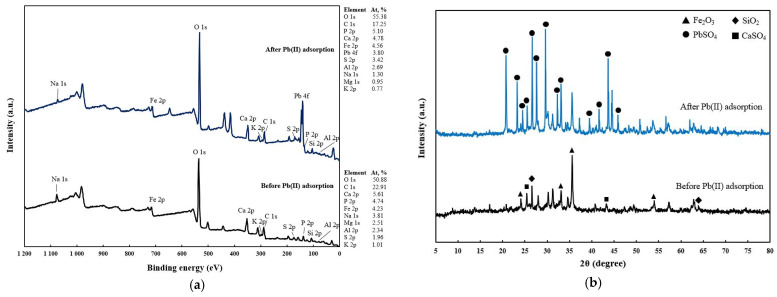
XPS spectra (**a**) and XRD patters (**b**) of nZVI30-ADSSBC-700 before and after Pb(II) adsorption.

SEM analysis was performed to study the surface morphology of nZVI30-ADSSBC-700 before and after Pb(II) adsorption at different magnifications. It can be seen that particles of ADSSBC pyrolyzed at 700 °C without N_2_ have an irregular shape and a rough surface (Figure 12a). The biochar surface is heterogeneous and contains numerous irregular cavities (Figure 12b). The EDS spectrum shows that the surface of nZVI30-ADSSBC-700 mainly contained C, O, Si, P, Ca, and Fe (Figure 12c). After Pb(II) adsorption, distinct Pb agglomerates were observed on the biochar surface, indicating successful Pb(II) adsorption (Figure 12d). A representative region containing Pb agglomerates is shown in Figure 12e. Tabular-shaped Pb structures were observed embedded within the pores of the biochar. The elemental composition of the Pb-rich region is shown in Figure 12f. The EDS spectrum confirmed the presence of Pb after adsorption, a finding that was further supported by XPS analysis (Figure 13a). The EDS spectrum also showed sufficient C and O content, which, together with Pb, could have contributed to the formation of lead carbonates, namely cerussite crystals [74]. In addition, sufficient Fe and O content was detected in this region, suggesting that Fe and O may have played a role in Pb(II) adsorption.

XRD spectra of nZVI30-ADSSBC-700 before and after Pb(II) adsorption are shown in Figure 13b. nZVI30-ADSSBC-700 showed diffraction peaks corresponding to quartz (SiO_2_) at 2θ = 26.6 and 63.9°, hematite (Fe_2_O_3_) at 2θ = 24.1, 33.1, 35.6, and 54.0°, and anhydrite (CaSO_4_) at 2θ = 25.4 and 43.2°. The presence of SiO_2_ is attributed to sand particles in sludge. Calcium-bearing phases likely originated from sludge liming during wastewater treatment. The formation of Fe_2_O_3_ in the biochar can be explained by partial oxidation of Fe species during pyrolysis under foil-wrapped conditions, where limited oxygen exposure occurred. After Pb(II) adsorption, diffraction peaks were observed at 2θ = 20.7, 23.3, 24.5, 25.5, 26.6, 27.6, 29.6, 32.3, 33.1, 39.5, 41.6, 43.7, and 45.9°, which were assigned to anglesite (PbSO_4_). This indicates that Pb(II) removal involved co-precipitation with SO_4_ species on the biochar surface. A similar observation was reported by Wu et al. [18], where municipal sludge-derived biochar pyrolyzed at 900 °C under N_2_ atmosphere showed CdSO_4_ formation after Cd(II) adsorption, confirming the role of sulphate in metal precipitation.

#### 3.11.2. Metal Ion Exchange

**Figure 14:**
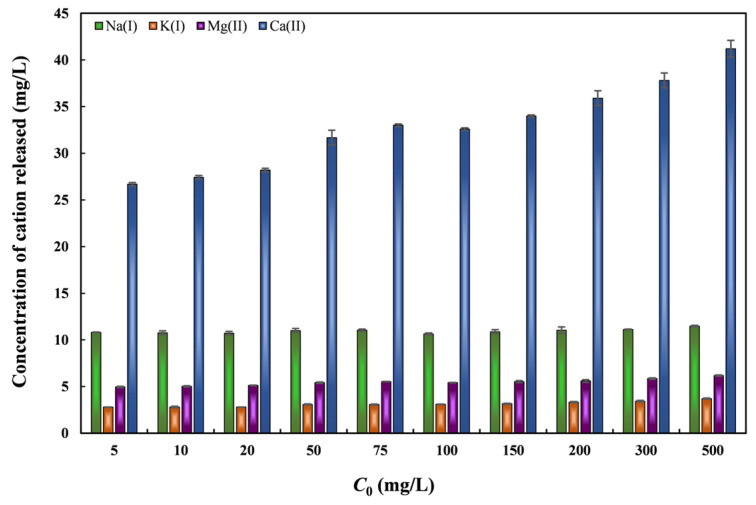
Variation in cation release with increasing initial Pb(II) concentration.

Ion exchange between ions present in the adsorbent and contaminant ions in solution is a typical process during adsorption. To evaluate this mechanism, the release of Ca(II), Mg(II), K(II), and Na(II) was measured after Pb(II) uptake (Figure 14). The results showed that nZVI30-ADSSBC-700 released the highest amount of Ca(II), with its concentration increasing consistently from 26.69 mg/L to 41.21 mg/L as the initial Pb(II) concentration increased from 5 to 500 mg/L, which was accompanied by a corresponding rise in Pb(II) *q*_e_ from 1.325 mg/g to 146.8 mg/g. To a much lesser extent, Mg(II), Na(I), and K(I) were involved in Pb(II) sorption. This indicates that Ca(II) ions play a major role in the ion-exchange mechanism. These results are in agreement with earlier research on biochar derived from ADSS, where the release of Ca(II) and Mg(II) from biochar was found to be related to the amount of adsorbed Pb(II) [14]. Ca(II) and Mg(II) preferentially occupy ion-exchange sites due to their charge similarity to Pb(II). This suggests that ion exchange plays a significant role in Pb(II) adsorption by biochar derived from digestates.

#### 3.11.3. Complexation

**Figure 15:**
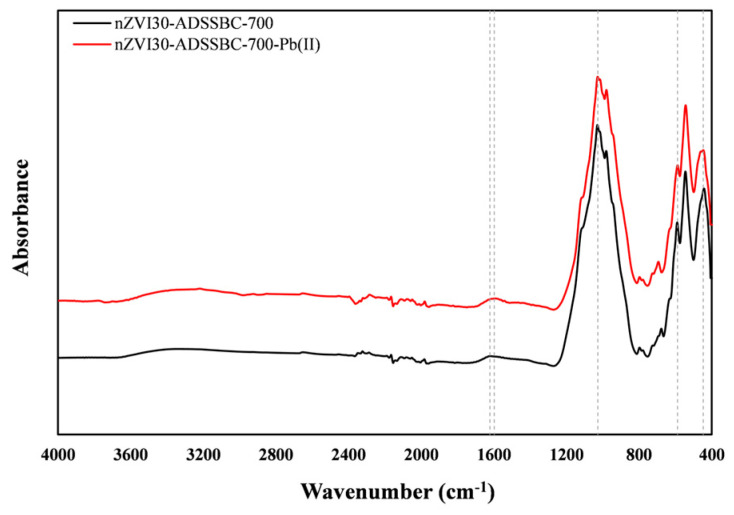
FTIR spectra of nZVI30-ADSSBC-700 before and after Pb(II) adsorption. The vertical dashed lines indicate the characteristic absorption bands discussed in the text.

The FTIR spectra of the studied biochar before and after Pb(II) adsorption are presented in Figure 15. The C=O peak shifted from 1622 cm^−1^ to 1598 cm^−1^ after Pb(II) adsorption, which could indicate the participation of the carbonyl group in Pb(II) complexation [75]. The band at 1030 cm^−1^, corresponding to C–O stretching vibrations, weakened after adsorption, further indicating that oxygen-containing groups were involved in the adsorption process. The small absorption peak at around 590 cm^−1^ may be associated with the Fe–O functional group, the intensity of which decreased after lead adsorption. This change could be related to the formation of internal Pb(II) complexes (Fe–O–Pb) during adsorption [76]. These changes observed after Pb(II) adsorption on biochar indicate a minor involvement of surface functional groups in Pb(II) binding. Consistent with minor changes observed in the FTIR spectra, complexation can be regarded as a secondary adsorption mechanism, while physical interactions may also contribute to the overall adsorption process.

**Figure 16:**
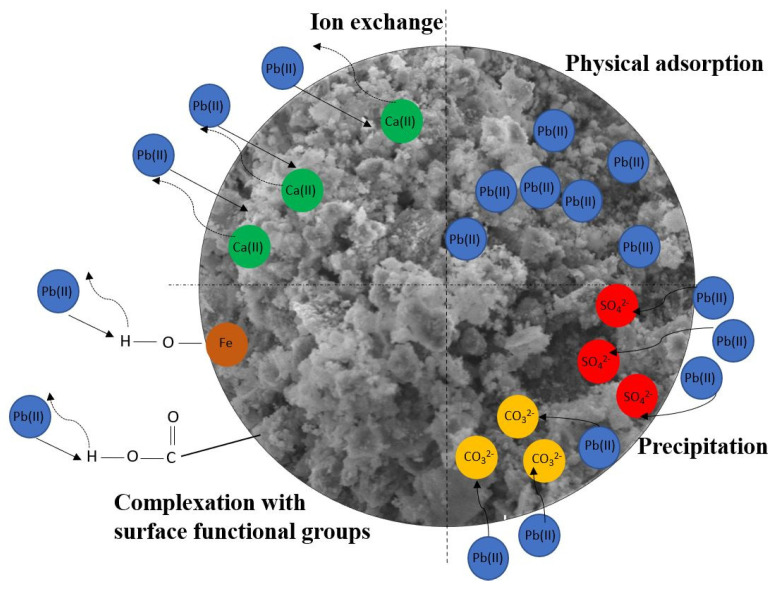
Proposed Pb(II) sorption mechanisms for nZVI30-ADSSBC-700.

The Pb(II) adsorption mechanisms on nZVI30-ADSSBC-700 are illustrated in Figure 16. Based on the SEM–EDS and XRD analyses and the changes in solution pH before and after the Pb(II) adsorption isotherm experiment, precipitation can be considered the dominant Pb(II) adsorption mechanism on nZVI30-ADSSBC-700, with PbCO_3_ and PbSO_4_ as the main precipitation products. Ion exchange may also have contributed substantially to Pb(II) removal, particularly involving Ca(II), as a positive correlation was observed between the increasing initial Pb(II) concentration and the concentration of leached Ca(II), as well as between Ca(II) release and Pb(II) adsorption capacity during the isotherm experiment. The results of FTIR analysis, as well as kinetic and thermodynamic modeling, confirmed that, in addition to chemical mechanisms, physical mechanisms were also involved in the adsorption process.

## 4. Conclusions

A modest increase in the Pb(II) adsorption capacity of biochar derived from ADSS was observed with increasing nZVI dosage applied to sludge before AD. Under foil-wrapped conditions, the adsorption capacity increased from 36.9 mg/g for biochar derived from non-pretreated ADSS to 41.9 mg/g with the application of 30 mg/g-TS nZVI prior to AD. The highest Pb(II) adsorption capacity (90.4 mg/g) was achieved by nZVI30-ADSSBC produced at 700 °C under foil-wrapped conditions. The adsorption behavior of Pb(II) was well described by the Langmuir and Freundlich isotherms and pseudo-first-order and pseudo-second-order kinetic models, with the Langmuir and pseudo-second-order models showing a superior fit. The maximum Langmuir adsorption capacity reached 139.3 mg/g, which exceeds the capacities of numerous sewage sludge-based adsorbents. Negative Δ*G*^0^ values from −4.98 to −7.63 kJ/mol and a positive Δ*H*^0^ value (21.34 kJ/mol) indicated that Pb(II) adsorption on nZVI30-ADSSBC-700 was spontaneous and endothermic, dominated by mixed chemical and physical adsorption mechanisms. SEM–EDS and XRD analyses revealed surface precipitation mechanisms, as evidenced by the formation of Pb-containing compounds on the biochar surface. Ion exchange was also involved, as indicated by the increased release of Ca(II) ions with increasing initial Pb(II) concentration. Furthermore, FTIR analysis indicated that complexation played a role in Pb(II) removal, as evidenced by the changes in the C=O, C–O, and Fe–O functional group peaks after Pb(II) adsorption. The disposal of spent biochar is a critical final step in sewage sludge management. Lead-loaded biochar can be treated with acids to remove adsorbed metal, with the residual Pb remaining stable. Alternatively, spent biochar can be incinerated for energy recovery. Following either treatment, biochar may be considered for potential use in construction materials.

## Figures and Tables

**Table 1:** Experimental conditions for the adsorption of Pb(II) on biochar derived from anaerobically digested sewage sludge pretreated with nZVI.

Type of Adsorption Experiment	Adsorbent Dosage, g/L	Initial pH	Contact Time *t*, min	Initial Pb(II) Concentration *C*_0_, mg/L	Temperature *T*, °C	Agitation Speed, rpm
Effect of nZVI dosage	2	4.9	360	200	22	12
Effect of pyrolysis temperature					20	
Effect of biochar dosage	0.2–4				23	
Effect of pH	2	1.0–5.0				
Effect of coexisting ions		4.9			24	
Effect of contact time			5–360		22	
Effect of initial Pb(II) concentration		4.8–5.4	120	5–500		
Effect of temperature		4.9		200	25–55	160

**Table 2:** Kinetic, isotherm, and thermodynamic models and parameters.

Models	Non-Linear Equation	Parameters	Reference
Pseudo-first order (PFO)	$q_{t}=q_{e}\times (1-e^{-k_{1}\times t})$,	*q*_e_—adsorption capacity (mg/g) at equilibrium, *q*_t_—adsorption capacity (mg/g) at time *t* (min), *k*_1_—rate constant of the PFO (min^−1^),	[44,45]
Pseudo-second order (PSO)	$q_{t}=\frac{{t\times k}_{2}\times q_{e}^{2}}{1+k_{2}\times q_{e}\times t}$,	*k*_2_—rate constant of PSO (g/(mg∙min)),	[45]
Intra-particle diffusion (IPD)	$q_{t}=k_{3}t^{1/2}+C$,	*C*—intercept of IPD model (mg/g), *k*_3_—IPD rate constant (mg/(g∙min^1/2^)),	[41,45]
Langmuir	$q_{e}=\frac{q_{max}\times K_{L}\times C_{e}}{1+K_{L}\times C_{e}}$, $R_{L}=\frac{1}{1+K_{L}\times C_{0}}$	*q*_max_—maximum adsorption capacity of Pb(II) (mg/g), *K*_L_—Langmuir adsorption constant, *C*_e_—concentration of Pb(II) at equilibrium (mg/L), *R*_L_—dimensionless separation factor, *C*_0_—initial concentration of Pb (II) (mg/L),	[39,41,46]
Freundlich	$q_{e}=K_{F}C_{e}^{1/n}.$	*K*_F_—Freundlich adsorption constant, 1/*n*—adsorption intensity,	[41,46]
Van’t Hoff	$lnK_{d}=-\frac{∆H^{0}}{RT}+\frac{∆S^{0}}{R}$, $K_{d}=\frac{q_{e}}{C_{e}}$, $∆G^{0}=-RTlnK_{d}$, $∆G^{0}=∆H^{0}-T∆S^{0}$.	$∆G^{0}$—Gibbs free energy (kJ/mol), $∆S^{0}$—entropy change (J/(mol∙K)), $∆H^{0}$—enthalpy change (J/mol), *R*—universal gas constant (8.314 J/(mol∙K)), *K*_d_—distribution coefficient of the adsorbate, *T*—absolute temperature (K).	[47]

**Table 3:** Kinetic fitting parameters for Pb(II) adsorption on nZVI30-ADSSBC-700.

Adsorption Model	Parameters	nZVI30-ADSSBC-700
PFO	*q*_e,cal_ (mg/g)	93.52
	*k*_1_ (min^−1^)	0.418
	*R* ^2^	0.9993
	*RMSE*	2.53
	*χ* ^2^	0.69
PSO	*q*_e,cal_ (mg/g)	95.21
	*k*_2_ (g/(mg∙min))	0.013
	*R* ^2^	0.9999
	*RMSE*	0.97
	*χ* ^2^	0.10
IPD	*K_diff_* (mg/(g∙min^1/2^))	2.41
	*C*	62.08
	*R* ^2^	0.9819
	*RMSE*	12.4
	*χ* ^2^	20.2
	*q*_e,exp_ (mg/g)	95.13

**Table 4:** Langmuir and Freundlich model parameters for Pb(II) sorption on nZVI30-ADSSBC-700.

Model	Parameters	Value
Langmuir	*K*_L_ (L/mg)	1.02
	*q*_max_ (mg/g)	139.3
	*R* ^2^	0.990
	*RMSE*	7.108
	*χ* ^2^	22.12
Freundlich	1/*n*	0.24
	*K*_F_ (L/mg)	49.49
	*R* ^2^	0.923
	*RMSE*	20.10
	*χ* ^2^	99.95

**Table 5:** Adsorption thermodynamics of Pb(II) on nZVI30-ADSSBC-700. Experimental conditions: biochar dosage = 2 g/L, initial pH = 4.9, *t* = 120 min, *C*_0_ = 200 mg/L, and agitation speed = 160 rpm.

*T* (K)	*q*_e_ (mg/g)	ln*K*_d_	Δ*G*^0^ (kJ/mol)	Δ*H*^0^ (kJ/mol)	Δ*S*^0^ (J/(mol∙K))
298.15	90.28	2.073	−4.984	21.34	88.28
308.15	91.23	2.216	−5.867		
318.15	92.24	2.499	−6.749		
328.15	93.29	2.859	−7.632		

**Table 6:** Pb(II) adsorption performance of different sludge-based adsorbents (ND—not determined).

Adsorbent	Pyrolysis Temperature (°C)	*C*_0_ (mg/L)	*q*_max_ (mg/g)	SSA (m^2^/g)	Reference
Magnetic nZVI-WSBC	600	400	206.5	ND	[63]
Biochar derived from anaerobic digestion sludge	600	200	51.20	ND	[15]
Pharmaceutical sludge-based biochar	500	2–20	19.07	ND	[7]
Biochar derived from sewage sludge and calcium sulfate	600	0–300	152.7	21.1	[5]
MSW-derived biochar treated with KOH	400	10–1000	106.3	3.0	[14]
Sludge-derived biochar	550	100–1000	30.88	24.7	[16]
Biochar derived from anaerobically digested sewage sludge pre-treated with nZVI	700	5–500	139.3	45.7	This study

## Data Availability

The raw data supporting the conclusions of this article will be made available by the authors upon request.

## Abbreviations

The following abbreviations are used in this manuscript:

SS	Sewage sludge
HMs	Heavy metals
ADSS	Anaerobically digested sewage sludge
SSA	Specific surface area
ADSSBC	Biochar derived from anaerobically digested sewage sludge
PFO	Pseudo-first-order kinetic model
PSO	Pseudo-second-order kinetic model
IPD	Intraparticle diffusion kinetic model
SD	Standard deviation
*RMSE*	Root mean squared error
TPV	Total pore volume
